# Preliminary nanopore cheminformatics analysis of aptamer-target binding strength

**DOI:** 10.1186/1471-2105-8-S7-S11

**Published:** 2007-11-01

**Authors:** Karen Thomson, Iftekhar Amin, Eric Morales, Stephen Winters-Hilt

**Affiliations:** 1 Department of Computer Science, University of New Orleans, New Orleans, LA 70148, USA; 2 Research Institute for Children, Children's Hospital, New Orleans, LA 70118, USA

## Abstract

**Background:**

Aptamers are nucleic acids selected for their ability to bind to molecules of interest and may provide the basis for a whole new class of medicines. If the aptamer is simply a dsDNA molecule with a ssDNA overhang (a "sticky" end) then the segment of ssDNA that complements that overhang provides a known binding target with binding strength adjustable according to length of overhang.

**Results:**

Two bifunctional aptamers are examined using a nanopore detector. They are chosen to provide sensitive, highly modulated, blockade signals with their captured ends, while their un-captured regions are designed to have binding moieties for complementary ssDNA targets. The bifunctional aptamers are duplex DNA on their channel-captured portion, and single-stranded DNA on their portion with binding ability. For short ssDNA, the binding is merely to the complementary strand of DNA, which is what is studied here – for 5-base and 6-base overhangs.

**Conclusion:**

A preliminary statistical analysis using hidden Markov models (HMMs) indicates a clear change in the blockade pattern upon binding by the single captured aptamer. This is also consistent with the hypothesis that significant conformational changes occur during the annealing binding event. In further work the objective is to simply extend this ssDNA portion to be a well-studied ~80 base ssDNA aptamer, joined to the same bifunctional aptamer molecular platform.

## Introduction and background

### Nanopore blockade detector

Our nanopore detector is biologically based and uses a protein, the α-hemolysin (α-HL) toxin produced by the bacterium *Staphylococcus aureus*, to create a pore through a phospholipid bilayer by self-assembly. The channel is selected due to its geometry and overall stability (i.e., minima gating), which allows molecules the width of dsDNA to be individually captured. A captured molecule reduces the observed ionic current in the channel, and the current level fluctuates as the molecule moves or binds. This fluctuating signal may "toggle" between more than one current level. An unchanging current reading that is lower than the open channel current value indicates the molecule is captured but not free to move. The values of the reduced current combined with the blockade level durations provide information about the captured molecule and its physical or kinetic properties.

**Figure 1:**
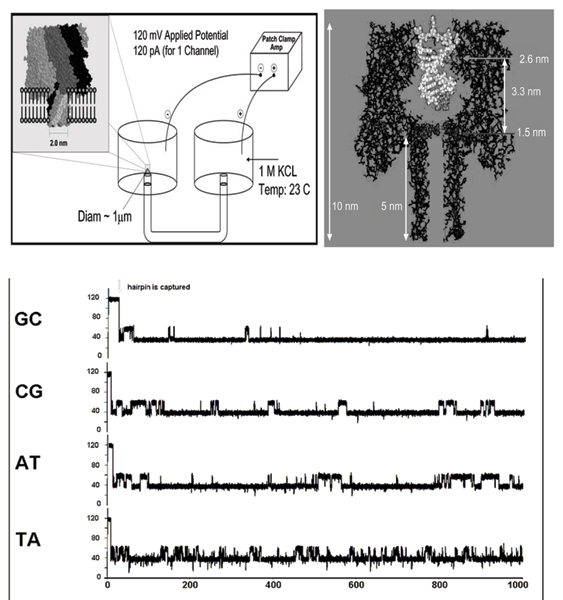
Left Panel: A lipid bilayer supports the alpha-hemolysin heptamer that creates the pore, or channel, used to collect the data, as shown left. The bilayer is established and supported across an aperture that typically provides 5–25 um in effective bilayer diameter, and generally greater than 1 um. Right Panel: The assembled alpha-Hemolysin pore shown to scale, with a captured dsDNA molecule. As shown, the double stranded form is too wide to pass through the pore, while a single strand may pass through. Bottom Panel: One-second blockade patterns of four DNA hairpins, part of a test set of nine base-pair hairpins, with 4 dT hairpin loops, that have been studied extensively. The molecules only differ in their terminal base-pairs, yet their channel current blockade signals, "signatures", are easily resolved [4].

**Figure 2:**
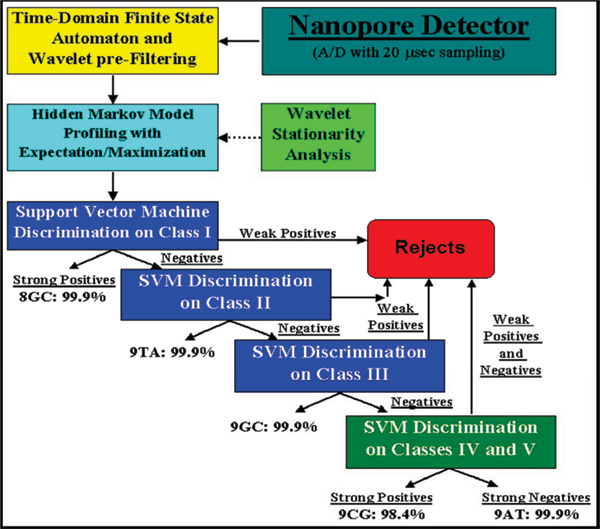
Classification software scheme and data flowchart. In real-time processing, the data from the detector is processed by wavelet FSA and stationarity analyses. From the HMM profiling, the SVM is used to classify the data, performing clustering analysis. The SVM is able to discriminate among known signal patterns with up to 99.9% accuracy

There are important distinctions in how a nanopore detector can function: direct vs. indirect measurement of static, stationary, dynamic (possibly modulated), or non-stationary channel blockades (see [1]). A nanopore-based detector can *directly *measure molecular characteristics in terms of the blockade properties of individual molecules – this is possible due to the kinetic information that is embedded in the blockade measurements, where the adsorption-desorption history of the molecule to the surrounding channel, and the configurational changes in the molecule itself directly, imprint on the ionic flow through the channel [2-7], see Figures 1 and 2. This approach offers prospects for DNA sequencing and single nucleotide polymorphism (SNP) analysis [7]. The nanopore-based detector works *indirectly *if it uses a reporter molecule that binds to certain molecules, with subsequent distinctive blockade by the bound-molecule complex. Such indirect observation of binding, or event transduction detection, is explored here in the case of DNA-DNA binding studies.

### Channel current cheminformatics

The signal processing architecture (Fig. 2) is designed to rapidly extract useful information from noisy blockade signals using feature extraction protocols, wavelet analysis, Hidden Markov Models (HMMs) and Support Vector Machines (SVMs). For blockade signal acquisition and simple, time-domain, feature-extraction, a Finite State Automaton (FSA) approach is used [8] that is based on tuning a variety of threshold parameters. A generic HMM can be used to characterize current blockades by identifying a sequence of sub-blockades as a sequence of state emissions [2-5,7]. The parameters of the generic-HMM can then be estimated using a method called Expectation/Maximization, or 'EM" [9], to effect de-noising. The HMM method with EM, denoted HMM/EM, is used in what follows (further Background on these methods can be found in [2-7]). Classification of feature vectors obtained by the HMM for each individual blockade event is then done using SVMs, an approach which automatically provides a confidence measure on each classification.

### Aptamers

Aptamers are essentially synthetically-derived, single stranded RNA or DNA molecules up to ~80 oligonucleotides in length with a high affinity towards bonding to specific targets. Consequently, aptamers may be ligands of proteins or other large biomolecules while having modified functional groups either during production or for added utility. In 1990, it was independently reported that a new method dubbed SELEX (Systematic Evolution of Ligands by EXponential Enrichment) provided a process of producing aptamers from random DNA or RNA libraries ([10] and [11]). Further development and application of real-time PCR in the production of aptamers has contributed to the growing effectiveness of aptamers in a variety of research areas today [12,13].

In a short number of years, there has been a growing preference for the use of aptamers over antibodies in a variety of different uses. The key to the success is how well aptamers do the same job in a diagnostic setting, as well as how versatile aptamers are in their design and usage. There is little to no conflict between design and function due to its origins in DNA and RNA, yet the structure is completely artificial. Since 1999, it has been reported that aptamers are being made in vivo as opposed to strictly in vitro [14], in addition to their use for in vitro diagnostics [15,16]. The main advantages of aptamers over antibodies are that aptamers are more durable (i.e., longer shelf life, do not require in vivo conditions, can sustain high immune response and toxins), more obtainable (i.e., cost effective, quicker to make, easily modified, uniformity due to synthetic origin), and have greater specificity and sensitivity (i.e., the degree of binding target recognition, lack of cross-species overlap) [12,13].

Aptamers may bind to multiple molecules simultaneously, thus allowing certain molecules or functional groups to bind more specifically, e.g., fluorescein, biotin bound to aptamers which target a specific protein. In fact, they can discriminate between small functional groups (i.e., methyl vs OH-, and D, L-enantiomers), and the specificity rivals that of antibodies and surpasses in some cases [17,13].

Properties of aptamers include the ability of a given structure to have multiple, stable, structural configurations (especially compared to peptides) due to Watson-Crick base pairing. Aptamers may bind with anything from dyes, drugs, peptides, proteins, metal ions, antibodies, and enzymes for example. The values of K_d _range between ~pML^-1 ^to ~nML^-1^, better than that of antibodies [18,19,13].

The production of DNA-based aptamers does not depend on the inclusion of particular functional groups [12] or a specific pH range to function [13], while such dependencies have been found for RNA aptamers. In fine tuning the production of RNA aptamers, it was found that various modifications made to RNA aptamers enabled closer sensitivity to buffer pH [20], metal ions and chelators [21,22].

Aptamers are now replacing antibodies as detection reagents due to having several advantages over antibodies: versatility, the creation of a lab-on-a-chip to process, low detection limits, simpler reactions to perform, diversity and specificity of aptamer-target binding properties [12]. The use of aptamer beacons has been used in flow cytometry [18], in place of antibody-based assays [12,17] and most abundantly in studies of specific proteins [15,16,19,23].

Since aptamers have high binding affinity and target specificity, there have been several investigations as to their structure and kinetic properties using techniques such as NMR and UV-Vis spectroscopy [24] but little is known about the binding behavior of such molecules on an individual level. Aptamers are appropriate for study by nanopore detection due to the fact they can be designed with an end to be captured by the nanopore (i.e., the captured end is dsDNA) while other parts of the aptamer are intended to bind with smaller aptamer components. The binding statistics derived from the study of aptamers in a nanopore detector can be used to provide insight into aptamer binding events. In future studies, the binding statistics for different aptamer pairs may be compared to derive design improvements of target binding affinity, among other characteristics. Improvements in binding affinity and specificity will improve the use of aptamers towards diagnostics, assays and pharmaceuticals, for example.

## Results

Experiments are performed with a linear molecule with a bulge in the center. To one side of the bulge is the blunt-ended stem sequence like that used in one of our DNA hairpin controls, where the bulge is now in the position of the hairpin's loop. To the other side of the bulge is a cap-section of base-pairs followed by an overhang section of length five bases. A similar set of experiments is performed with the "Y-aptamer", a Y-shaped DNA complex with one arm of the Y with an overhang (6 Ts), while the other arm is capped with a 4 dT loop. The base of the Y is a stem of 10 base-pairs length, prior to the Y-nexus of the molecule. Here the Y-nexus is in the place of the bulge, or the hairpin loop. Nine or ten base-pairs is approximately the length in dsDNA from the mouth of the channel to its limiting aperture. The significance of this length in the modeling is due to its delicate placement of the end of the captured molecule over the high electrophoretic field strength zone near the limiting aperture of the channel, permitting operation in transduction mode [1]. The overhang's binding strength can be adjusted by tailoring its length in both of these experiments, and in future work this will also permit a highly precise study of DNA annealing.

**Figure 3:**
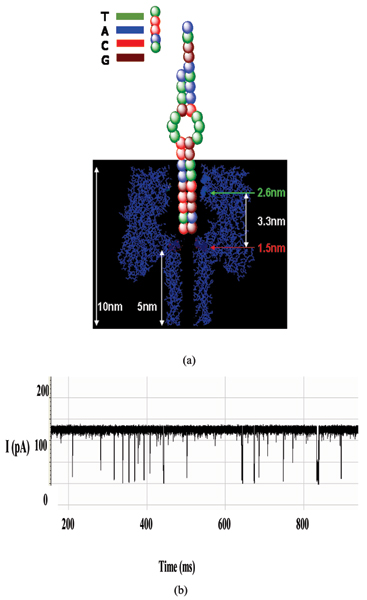
Translocation of a single DNA strand. (a) A crude molecular schematic illustrating a captured pseudo-aptamer before binding to the 5 nucleotide overhang complement. If the two strands were to separate, the strands would pass through the vestibule one strand at a time, as an aptamer melting event and ssDNA translocation. (b) Raw data shows the ssDNA translocation signals (most involving molecules that are already single-stranded upon capture).

**Figure 4:**
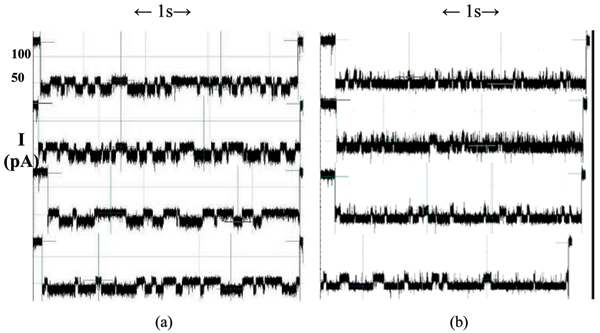
(a) Collection of toggle signals from the captured pseudo-aptamer. The molecule appears as the one in Fig. 3(a), unattached to the 5 nucleotide ssDNA. (b) Collection of toggle signals from the pseudo-aptamer + 5 nucleotide ssDNA. The bound complex appears as in Fig. 3(a), with the 5 nucleotide sequence joined to the overhang section. One distinctive change only observed in the blockade signals after binding agent introduced, aside from the level dwell-time changes, are the much higher frequency upward "spike" transitions, from the lower level to the upper level.

**Figure 5:**
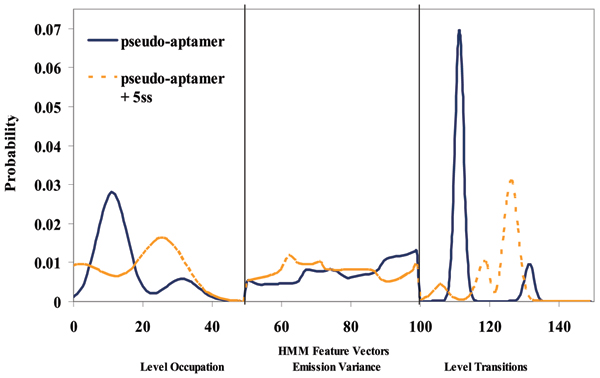
Level duration feature extraction for the 6 T pseudo-aptamer. First feature extraction for the pseudo-aptamer bound to its complementary 5 base pair sequence. The y-axis is the transition probability, while the x-axis is the HMM Profile: 150 feature vectors components are obtained from the 50-state HMM-EM/Viterbi implementation in [4] are: the 50 dwell percentage in the different blockade levels (from the Viterbi trace-back states), the 50 variances of the emission probability distributions associated with the different states, and the 50 merged transition probabilities from the primary and secondary blockade occupation levels (fits to two-state dominant modulatory blockade signals).

The linear duplex DNA molecule, with bulge, and ssDNA overhang, is shown captured by the channel in Figure 3. Examples of the signals that occur when a properly annealed duplex is captured are shown in Fig. 4. Figure 5 compares signal traces before/after in terms of their standard 150-component feature set (see Methods and [4]).

**Figure 6:**
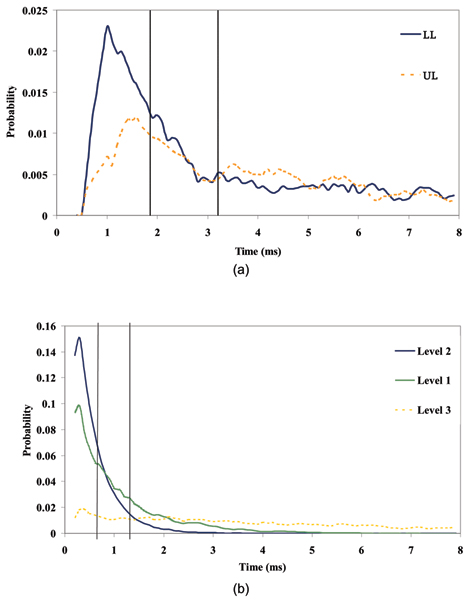
(a) Dwell-time distributions in the dominant blockade levels for the pseudo-aptamer. The vertical dotted lines indicate the median dwell time for two levels. (b) Same analysis for the pseudo-aptamer bound to its corresponding 5 base pair sequence, with the emergence of third level not present in the unbound pseudo-aptamer. The two vertical dotted lines indicate the median dwell times for the first and second levels, while the third level median dwell time value extends to 9.54 ms (plot scale has been truncated for space).

Using our HMM-based cheminformatics software, we can determine the median values of the dwell time per level. The dwell time corresponds to the interval of time before transition to another level. A capture signal has noticeably different current value (between ~20–70 pA) than the open channel current (~120 pA). A blockade (or toggle) will transition between two or more current values, given the difference between the levels is greater than the signal noise level. (Notation: the assignment of levels is determined by increasing mean current value. For example, the lowest level may have a mean current around 35 pA, the intermediate level around 40 pA, and the upper level around 55 pA.) The median value can be interpreted as the half-life in biophysics terms or as ~1/k_off _in biochemical terms. The binding constant K_d _is proportional to k_on_/k_off_. Figure 6 shows the dwell-time statistics for the dominant blockade states.

**Figure 7:**
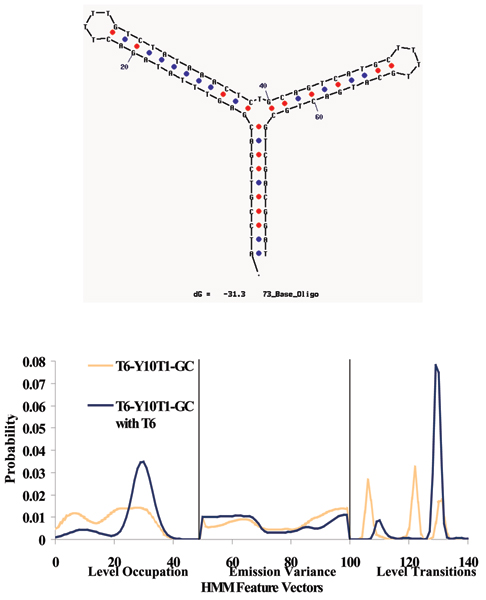
Standard 150-component HMM-based feature extraction for the T6-Y10T1-GC (mfold diagram for molecule shown top), and for the T6-Y10T1-GC +T6 Y-aptamer blockade signals. (See Fig. 5 Caption for explanation of HMM features.)

**Figure 8:**
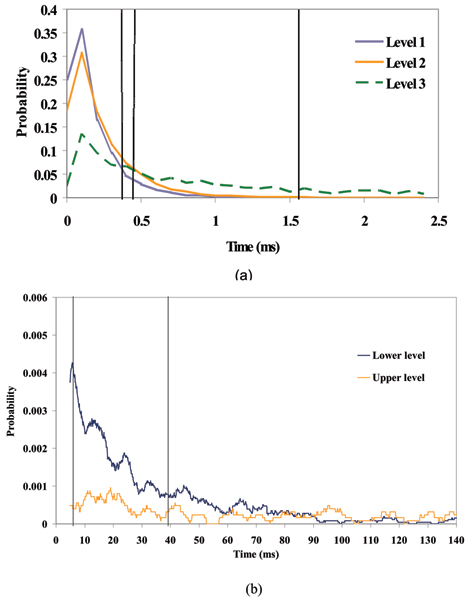
The dwell times at each level for the Y-aptamers. (a) Kinetic feature extraction associated with analysis of the signal shown in Figure 7 for the Y-aptamer T6-Y10T1-GC. The vertical dotted lines stand in place of the median dwell times. (b) Same analysis for the Y-aptamer bound to the additional T6 appendage, with the disappearance of third level present in the unbound Y-aptamer. Again, the vertical lines stand in place of the median dwell times for each level.

For the unbound pseudo-aptamer, the median values of these distributions are 1.78 ms and 3.12 ms (lower, upper levels respectively – see Fig. 6a). For the pseudo-aptamer + 5ss DNA (whose 150-feature profile is shown in Fig. 7), the median values of the distributions are 1.26 ms, 0.72 ms and 9.54 ms (first, second and third levels respectively – see Fig. 6b). Besides the pseudo-aptamer, the Y-aptamer complex T6-Y10T1-GC was found to have mean dwell time values of 0.30 ms, 0.38 ms and 1.52 ms (for the first, second and third levels respectively – see Fig. 8a). When a T6 group was added to bind with the T6-Y10T1-GC to form a T6-Y10T1-GC+T6 complex, the mean dwell time statistics differed significantly, becoming dominated by fewer blockade levels, consistent with the annealed molecule "stacking" into the duplex helix, thereby having fewer accessible degrees of freedom. The bound complex has a lower level mean dwell value of 3.62 ms, while the upper level has the value of 39.14 ms (the difference as indicated by Fig. 8b).

## Discussion/conclusion

### Pattern recognition informed feedback via LabWindows automation

The blockade stationary statistics or phases thereof, reveal information about the kinetics of the biopolymer resulting from interactions with another molecule, from interactions with the channel, or from undergoing conformational changes. LabVIEW Automation software is used to help manage the feedback linkage between patch-clamp amplifier measurements and in-house cheminformatics software. This has been used to do demonstrate molecular identification in the first 100 ms of capture, with return of classification information to the control of the amplifier – for voltage-controlled sample ejection if desired [25]. The nanopore system, thus, offers the remarkable prospect of providing a single molecule view of individual molecular interactions.

It is important to remember that the statistics obtained are for a single molecule, as opposed to a large collection of molecules. While diagnostic methods which employ aptamers in a dye-quenching process involve thousands if not millions of molecules (in a small volume) to obtain statistically significant results, the signal acquired by the nanopore detector is dependent on the statistics of small numbers. The process of detecting aptamer-beacons takes only a short while due to the large numbers of molecules contributing to the overall signal. If the diagnostic were carried out with a single fluorescing molecule bound to an aptamer, the photons absorbed and emitted would be few and far between, resulting in data completely buried by noise. Therefore, the statistics determined by the analysis of a single molecule are a snapshot of the conditions present at the time of data collection, as opposed to averaging numerous signals collected over time together.

This brings up the discussion of the fundamental physics involved in the examination of a single molecule. Does a single biomolecule behave in a way that complies with classical or quantum mechanics? A collection of gas molecules can be classical, while a single gas molecule emits radiation generally according to allowed transitions in quantum mechanics. Classical or quantum can affect the method of data collection and resulting statistical analysis. Our analysis of a single aptamer (and larger biomolecules) so far remains in the classical regime, since the molecules are thoroughly decohered in their quantum phase, immersed as they are in a highly interactive, stationary, ionic flow. If the data of interest were a measure of change occurring when an attached dye emitted or absorbed a photon, however, the measurements utility might be limited by quantum statistical noise.

The time-scale for the level-switch, or molecular state-changes, is clearly different before and after introduction of the complementary 5ss DNA binding partner. Furthermore the dominant levels increase from two to three, indicating a large-scale change to the molecule, possibly due to conformational change in the molecule itself upon binding. Similar observations hold for the annealing experiments with the Y-aptamer and its 5'-TTTTTT-3' overhang. Refinements of single-molecule binding information like this will lead to a much more informed aptamer design process. For the moment, a prototype of this informed aptamer design process is simply in the selection for clearer observation of on/off binding: Right now we see clear examples of on-binding (with a variety of controls to reassure us that that is what we are seeing), but nothing conclusive has been done with observation of off-binding. Once we can get an annealing or other binding interaction with k_on _and k_off _statistics, we can pursue a number of fundamental experiments as to the nanopore detectors overall capabilities in this regard. The ability to observe binding, and select for binding strength of interest, could aid in the identification of tumor-specific molecular markers, for example, which is a powerful tool in cancer diagnostics, and targeting of tumor specific pathways is the best hope for developing non-toxic and efficient anti cancer therapies. Targeting of cancer cells relies on the development of molecules, aptamers with toxins in tow, perhaps, that are suited to in vivo applications and that have the required affinity, specificity and favorable pharmacokinetic properties.

## Methods

### Nanopore experiments

Each experiment is conducted using one α-hemolysin channel inserted into a diphytanoyl-phosphatidylcholine/hexadecane bilayer across a 25-micron-diameter horizontal Teflon aperture, as described previously [4,7] (Fig. 1). The α-hemolysin pore has a 2.0 nm width allowing a dsDNA molecule to be captured while a ssDNA molecule translocates. In Figure 1, the effective surface area of the bilayer ranges between 5–25 μm. This value has some fluctuation depending on the condition of the aperture, which station is used, and the bilayer applied on a day to day basis. Seventy microliter chambers on either side of the bilayer contain 1.0 M KCl buffered at pH 8.0 (10 mM HEPES/KOH) except in the case of buffer experiments where the salt concentration, pH, or identity may be varied. Voltage is applied across the bilayer between Ag-AgCl electrodes. DNA control probes are added to the *cis *chamber at 10–20 μM final concentration. All experiments are maintained at room temperature (23 ± 0.1°C), using a Peltier device.

### Control probe design

The five DNA hairpins studied in the nanopore prototype experiment [4] have been carefully characterized, and are used here as highly sensitive controls. The nine base-pair hairpin molecules examined in the prototype experiment share an eight base-pair hairpin core sequence, with addition of one of the four permutations of Watson-Crick base-pairs that may exist at the blunt end terminus, i.e., 5'-G•C-3', 5'-C•G-3', 5'-T•A-3', and 5'-A•T-3'. Denoted 9GC, 9CG, 9TA, and 9AT, respectively. The full sequence for the 9CG hairpin is 5' CTTCGAACGTTTTCGTTCGAAG 3', where the base-pairing region is underlined. The eight base-pair DNA hairpin is identical to the core nine base-pair subsequence, except the terminal base-pair is 5'-G•C-3'. The prediction that each hairpin would adopt one base-paired structure was tested and confirmed using the DNA mfold server .

### DNA probe construction and testing

The linear aptamer with bulge consists of annealing the following two ssDNA strands:

(1) 5'-GAGGCTTGG TTT CAATAGGTA-3'

(2) 5'-ATTG TTT CCAAGCCTC-3'

The overhang region has sequence (3) as part of (1). The complementary 5 nucleotide ssDNA sequence (4):

(3) 5'-AGGTA-3'

(4) 5'-TACCT-3'

Likewise, the Y-aptamer DNA molecule consists of a three-way DNA junction created by annealing two DNA molecules:

(1) 5'-CTCCGTCGAC GAGTTTATAGAC TTTTTT-3'

(2) 5'-GTCTATAAACTC GCAGTCATGCTTTTGCATGACTGC GTCGACGGAG-3'

For the resulting Y-aptamer, one of the junctions' arms terminate in a 4 dT-loop and the other arm has a 6 T overhang in place of a 4 dT-loop. The blunt ended arm has to be carefully designed such that when it is captured by the nanopore it produces a toggling blockade. One of the arms of the Y-shaped aptamer (Y-aptamer) has a TATA sequence, and is meant to be a binding target for TBP. In general, any transcription factor binding site could be studied (or verified) in this manner. Similarly, transcription factor could be verified, or the efficacy of a synthetic transcription factor could be examined.

### HMM construction and feature extraction profiles

The features extracted for each blockade signal are as follows: the first 50 components are the level occupation probabilities for the blockade signal in the range 20% to 70% of the open channel current (with bin size at 1%). The blockade signal entirely resides in this blockade range, which is minimized for faster HMM processing. The middle 50 components are the variances of the emission probabilities for each of the 50 blockade levels (normalized as an L1 vector). The last 50 components shows a compression of the 2500 transition probabilities to the 50 values obtained from merging the transition probabilities from the two strongest levels indicated in the analysis of the first 50 components (with weighting as indicated by their support in the level occupation probabilities). The software is accessible via a web interface at , see [25] for latest discussion of the web interface. The analysis tools have the ability to "zoom" into areas of interest, usually maintaining the 50-state HMM, but with bin size reduced accordingly, to focus on the "zoomed" range of interest. In this analysis, level fine-structure is not sought, and the 150-component profiles are easily discernible by eye, such that smaller, "zoomed", bin-sizes for greater clarification aren't needed.

## Competing interests

The authors declare that they have no competing interests.

## Authors' contributions

The initial submission was written by KT and SWH, with revisions by SWH. Most of the data was gathered by KT after being trained on nanopore detector operations by IA and EM. IA and EM also helped in gathering the data. EM helped in constructing the nanopore device. Experimental design and pattern recognition software contributed by SWH.
